# The changing *ethnoecologicalcobweb* of white truffle (*Tuber mangnatum* Pico) gatherers in South Piedmont, NW Italy

**DOI:** 10.1186/s13002-016-0088-9

**Published:** 2016-04-18

**Authors:** Andrea Pieroni

**Affiliations:** University of Gastronomic Sciences, Piazza Vittorio Emanuele 9, I-12060 Pollenzo, CN Italy

**Keywords:** Ethnomycology, Ethnoecology, Truffles, Piedmont, Italy

## Abstract

**Background:**

Traditional Environmental Knowledge (TEK) related to truffles represents an under-investigated area of research in ethnobiology. Nevertheless, truffles, in a few southern European areas, and notably in South Piedmont, represent a crucial component of the local economy and cultural heritage.

**Methods:**

Thirty-four white truffle (*Tuber magnatum* Pico) gatherers, locally known as *trifulau*, aged between 35 and 75 years and living in a few villages and small towns of the Langhe and Roero areas (South Piedmont, NW Italy), were interviewed in-depth during the years 2010-2014 regarding their ecological perceptions, truffle gathering techniques, and the socio-ecological changes that have occurred during the past several decades.

**Results:**

A very sophisticated ethnoecological knowledge of the trees, soils, and climatic conditions considered ideal for searching for and finding white truffles was recorded. Moreover, a very intimate connection between gatherers and their dogs plays a fundamental role in the success of the truffle search. However, according to the informants, this complex *ethnoecological cobweb* among men, truffles, dogs, and the environment has been heavily threatened in the past few decades by major changes: climate change, in which the summer has become a very hot and dry season; social changes, due to a more market-oriented attitude of younger gatherers; and especially environmental and macro-economic dynamics, which followed the remarkable expansion of viticulture in the study area.

**Conclusion:**

The TEK of white truffle gatherers indicates the urgent need for fostering sustainable gastronomy-centred initiatives, aimed at increasing the awareness of consumers and food entrepreneurs regarding the co-evolution that has inextricably linked locals, truffles, and their natural environment during the past three centuries.

## Background

Studies focusing on Traditional Environmental Knowledge (TEK) systems concerning food have become important in the last decade to promote endogenous strategies for sustaining food security and sovereignty [1, 2]. Folk knowledge systems concerning *foodscapes* (foods and their environments) are in fact important for contributing to long-term sustainable gastronomies, since these systems represent the result of a long co-evolution between local communities and their *oikos*; in other words, TEK represents one of the most potent ways through which socio-ecological systems exercise their resilience over time.

Although ethnomycological knowledge is well documented in different parts of the globe, especially in Asia, Africa, and Central America [3–14], folk knowledge concerning food fungi has rarely been taken into account in Europe, with the exception of a few studies conducted in Eastern Europe [15–18] and some sporadic work carried out in Italy [19, 20]. Truffles, which are hypogeous mushrooms, have been the focus of very few ethnobiological studies, mainly in desert areas [21–24], while in the environmental anthropological literature the folk knowledge systems of European truffle gatherers, their perceptions of the environment, as well as their dynamics over time have been little researched [25–27].

An ethnobiological study regarding truffle gatherers in a culturally crucial territory for the truffle culture like South Piedmont could therefore offer an important contribution for understanding locals’ perceptions of this neglected domain. Truffle gatherers (*trifulau* in Piedmontese) have possibly accumulated and organized a unique understanding of their natural environment over generations and for this reason their perceptions of the ecosystem over time are important for the sustainability of this practice and worth investigating.

The aims of this study were therefore:

- to document truffle gatherers’ folk knowledge on ideal soil, trees, and climatic conditions for searching for white truffles;
- to document collectors’ knowledge of truffle gathering techniques, and especially the link between dogs and truffle gatherers, as well as the mechanism of transmission of this knowledge;
- to document possible perceptions regarding socio-ecological changes related to traditional truffle gathering experienced during the past decades.

## Methods

### Environmental and social backgrounds

**Fig. 1:**
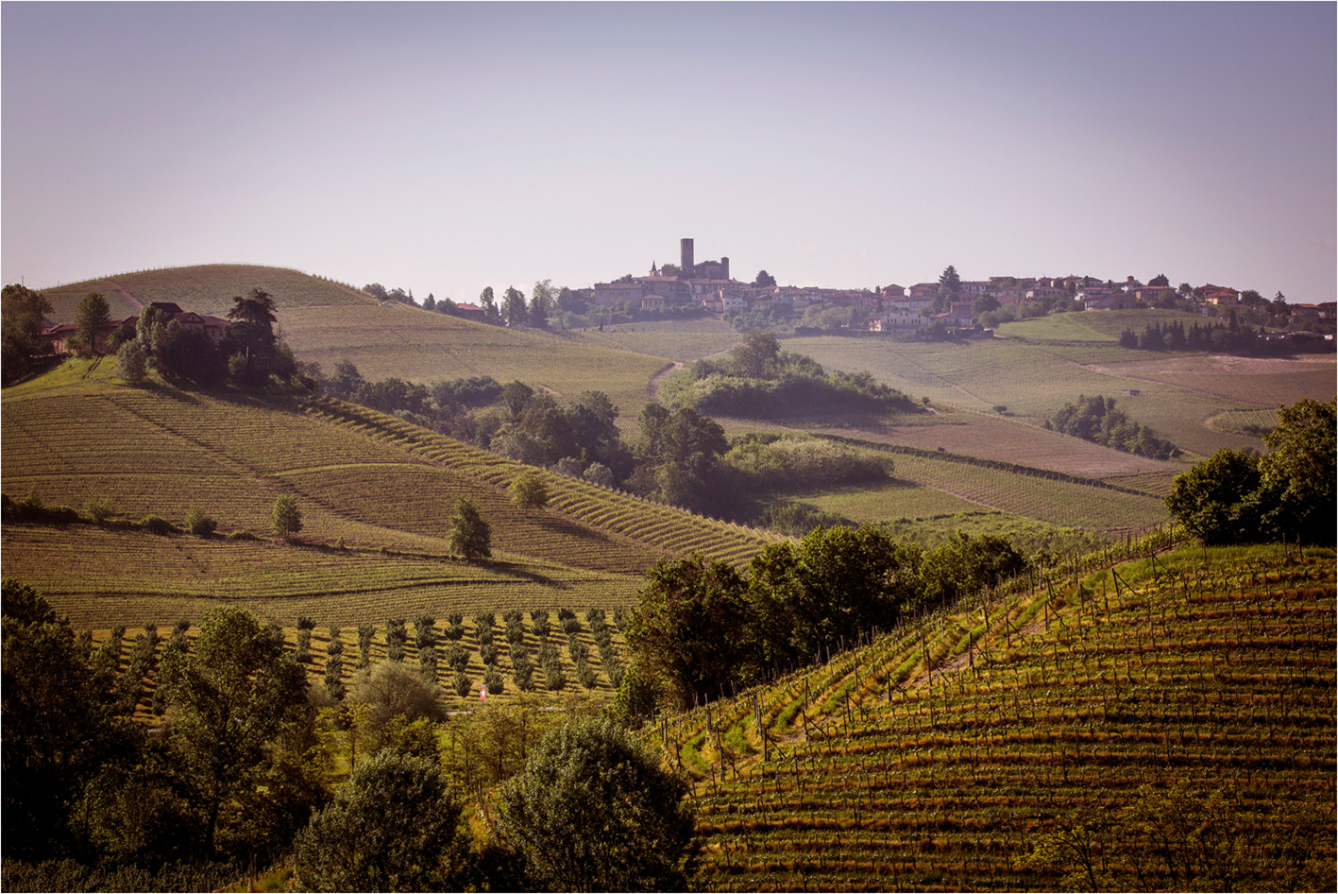
Landscape of Langhe (courtesy of Marcello Marengo, University of Gastronomic Sciences, Pollenzo, Italy)

Langhe and Roero are more or less hilly areas in South Piedmont, NW Italy, famous for their prized wines (especially Barolo) and stunning landscape, which is the result of a long interaction between locals and nature, as vineyards first appeared in the 2^nd^ half of the 18^th^ Century (Fig. 1). Together with the vineyards, the landscape presents some cultivations of hazelnut and poplar trees, and a few vegetable plots, where mainly sugar beets (for fodder) and other vegetables (notably local cultivars of sweet peppers and leeks) are cultivated.

The secondary forest mainly consists of pubescent oak (*Quercus pubescens* Willd.) and other oak species, wild hazelnut trees (*Corylus avellana* L.), Cornelian cherry trees (*Cornus mas* L.), ash trees (*Fraxinus excelsior* L.), and wayfarers (*Viburnum lantana* L.), while the hedges are home to osier (*Salix viminalis* L.), dog rose (*Rosa canina* L.), hawthorn (*Crataegus* spp.), privet (*Ligustrum vulgare* L.), and honeysuckle (*Lonicera caprifolium* L.).

In this ecosystem, the search for wild white truffles (*Tuber magnatum* Pico) during late autumn is a crucially important part of the culinary and social history of the local gastronomy; in particular, the former Piedmontese Royal House (and, later, Italian Royal House) helped to promote the virtues of local truffles among diverse European courts beginning in the 18^th^ Century [28–31]. The white truffle from South Piedmont (also known as *tartufo bianco di Alba -* Alba’s white truffle) is one of the most expensive and most appreciated food products in the gastronomic world, both inside and outside of Italy. Together with Barolo wine, it has gained worldwide recognition as a symbol of the high-quality South Piedmontese gastronomy, and wealthy food lovers are ready to pay even 500 Euros for a hundred grams.

According to Italian media, the yearly revenue of the white truffle market in Italy is estimated to be around 400 million Euros, of which the largest portion is represented by the South Piedmont harvest. These are estimations, however, since the white truffle market still predominantly moves in a “grey” area, in which transactions among gatherers, intermediaries, and restaurants owners often take place under the table. Moreover, it is crucial to underline the new phenomenon of private gatherers who sell online, via eBay for example, their freshly collected white truffles, especially in Southern areas of Italy, where, despite some availability of the product in the wild, the luxury food market for truffles (high-quality restaurants, foreign tourists) is missing.

White truffles from Alba are the highlight of every *gourmet* menu in the study area from October to January, and the way they are presented in the few truffle shops (devoted almost exclusively to tourists) can be compared with the exhibition of an expensive piece of jewellery. However, the Piedmontese truffle culture consists of more than what the bright lights of the Alba Truffle Fair, which takes place mid-October every year, illuminate. For most tourists, who enjoy slices of fresh white truffles with buttered egg noodles, poached eggs, cheese fondue, or raw ground beef in local restaurants, the part of story which happens before the fragrant hypogeus fungus finds its way into these local dishes is largely unknown. The culture and history behind truffles remain under the surface in most cases, although, according to the local truffle gatherers associations, there are currently around 10,000 officially registered truffle gatherers (better known in the local Piedmontese language/dialect as *trifulau,* both in singular and plural), i.e. people who possess a licence for searching for truffles, which is mandatory according to national and regional laws.

### Field study

**Fig. 2:**
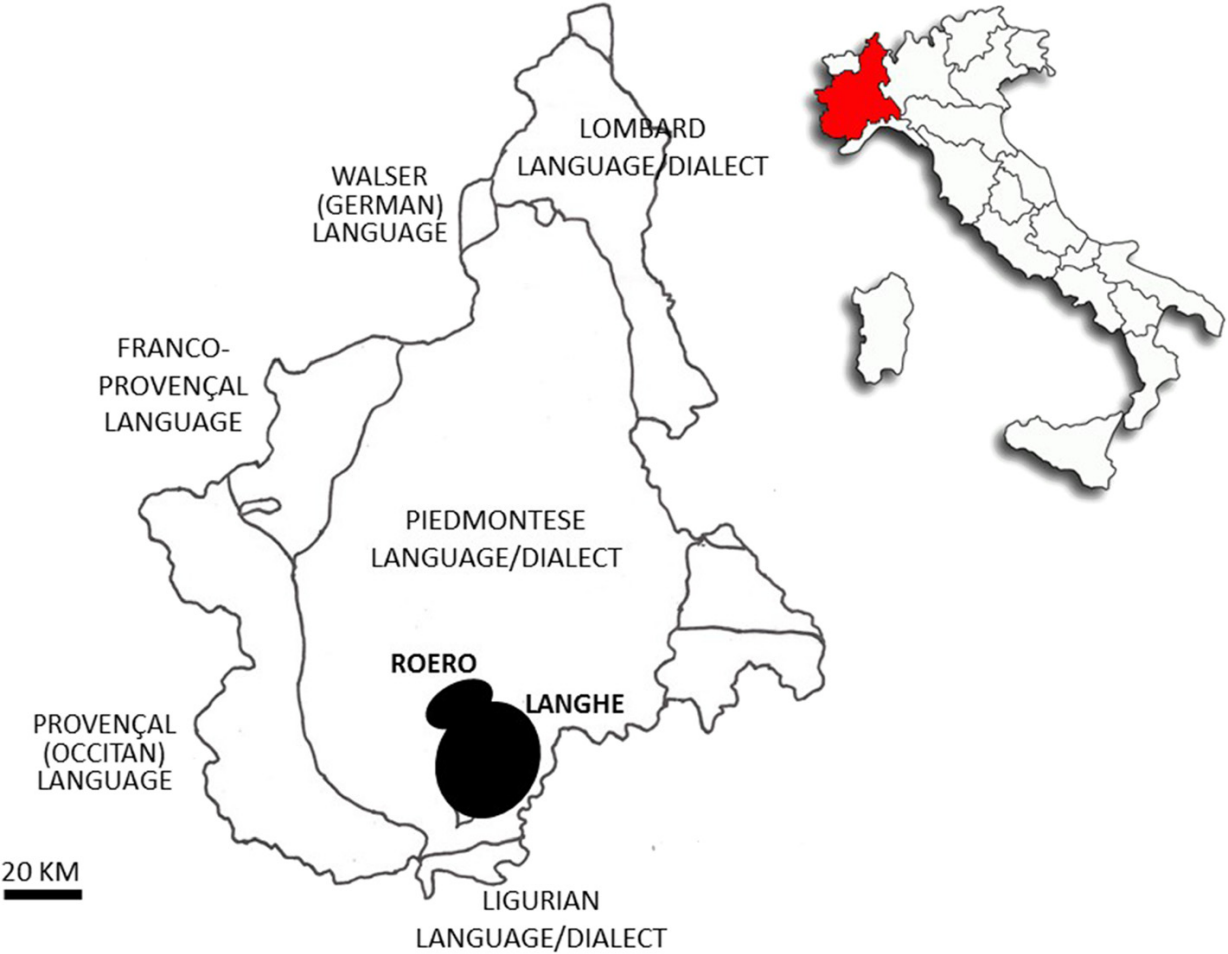
The study areas within the linguistic map of Piedmont, NW Italy

During the years 2010-2014, in a few villages and small towns of the Langhe and Roero areas (South Piedmont, Fig. 2), semi-structured interviews with thirty-four male *trifulau* between the ages of 35 and 75 years (average age: 62), who were identified and selected using snowball sampling techniques, were conducted.

The interviews focused on their TEK of the *oikos* where white truffles grow, their truffle gathering techniques, and changes that have occurred between their late childhood and today. With a few selected *trifulau*, in-depth interviews specifically addressing the mechanisms of transmission of TEK related to truffles were also conducted. Prior Informed Consent was always obtained before each interview and the Code of Ethics of the International Society of Ethnobiology was strictly followed [32]. The interviews were conducted in standard Italian and, more rarely, in the Piedmontese dialect and sometimes audio or video recordings were taken, if the study participants expressly agreed to this. A part of the collected video material is stored in the University of Gastronomic Sciences visual ethnographic database [33].

## Results

### *Trifulau* and their perceptions of trees, soils, and climate

The interviewed *trifulau* were all males, in their middle or third ages. For all of them truffle gathering is, or has been, a secondary occupation and a supplementary source of income. Their occupations were in fact quite diverse and varied from being retired, to construction workers, clerks, and industrial managers. When asked about the gender imbalance, the *trifulau* seemed surprised and confirmed that traditionally only men have been gatherers, even though the main expertise in using truffles in the local cuisine (mainly sliced raw on top of egg-based dishes) is a specific skill of women. A few gatherers’ wives, who were interviewed together with their husbands, also commented – not without some irony – that consuming white truffles would act as a male aphrodisiac.

*Trifulau* generally gather diverse species of truffles throughout the year, especially black (locally known as “sweet”, *Tuber melanosporum* Vittad.) and summer truffles (*Tuber aestivum* Vittad.), but the collection of white truffles only and the white truffle season (late autumn) are considered by all the pillar and peak of their activity. This is due to the overwhelming prestige, smell, rarity, and price of the white truffle, as well as the sense of Piedmontese identity that the white truffle is able to evoke, which cannot be compared with the gathering of other local tuber species.

A few *triflulau* even stated that they do not waste time with other truffles, and that they only search for white truffles during late autumn. All interviewees expressed a very close affective connection to nature and especially to the secondary forests and transitional patches covered by trees at the edge of cultivated fields, where truffles may grow. The respondents mentioned the love or passion for nature that is required to be successful in the search for truffles. Repeatedly, this attribute was referred to as something that cannot be learned and that is innate, or at least connected to growing up in the countryside. According to the respondents, a *trifulau* must be able to understand and interpret various natural information and processes.

Seasons and the ability to evaluate and predict weather conditions in different areas are playing a crucial role as different temperatures and climatic conditions are not only increasing or decreasing the chances of finding truffles but also influencing their quality and ultimately their price on the market. November and December for example, when temperatures are below freezing at night, are generally considered ideal months for white truffle harvesting. The better the *trifulau* knows his territory and its natural characteristics, the better he is able to identify suitable gathering places depending on weather and soil conditions. Many *trifulau* also see themselves as guardians of the ecological system surrounding them. Table 1 documents the perceptions a few *trifulau* have regarding the most ideal ecological conditions for the growth of white truffles.

**Table 1:** Gatherers’ perceptions of the optimal periods and environmental conditions for the growth of white truffles

Informant	Gathering period	Trees	Soils	Climate
1	Mid Sept-mid Jan; best time however is in November	Elm (*Ulmus minor* Mill.), Willow (*Salix alba* L.), Birch (*Betula pendula* L.), Oak (*Quercus* spp.)	Sandy soils “produce” round truffles; muddy soils flat and irregular truffles	Rain at the end of June-beginning of July is crucial for a good gathering season
2	Oct-Jan	Oak, Poplar (*Populus* spp.), Willow	Wet and ventilated soils; floods can stop the growth of truffles for many years	In July and August it must rain; snow in the gathering period does not have any influence
3	Mid Sept-mid Dec	Poplar, Lime Tree (*Tilia platyphyllos* Scop.), Dog Rose (*Rosa canina* L.), Cornelian Cherry Tree (*Cornus mas L*.), Willow, Pubescent Oak (*Quercus pubescens* Willd.)	Acidic soil; landslides stop the growth of truffles for a few years	Summer droughts generate very little growth in the fall
4	Aug-Jan	Elm, Oak, Birch, Willow	Wet and sandy soils; landslides and floods are very detrimental	Landslides and floods are very dangerous for the growth of white truffles, although they may be useful as well, since they shift and “reorganise” the occurrence of truffles elsewhere
5	Oct-Dec	Osier (*Salix viminalis* L.)	Pesticides and floods are very detrimental	Fresh and wet climate
6	Oct-Dec	Oak, Poplar, Hazelnut (*Corylus avellana* L.), Willow, Lime Tree	Soil exposure along the North-South direction	Wind occurrence
7.	End of Aug-beginning of Dec	Willow, Oak, Hazelnut	Fresh and wet soils	
8	Aug-Dec	Poplar, Willow, Oak, Dog Rose	“Heathy” soils – a sign of these is the occurrence of many snails	Wet climate
9	Sept-Nov	Elm, Lime Tree, Pubescent Oak	Wet and fresh soils; windy slopes exposed to the North	Ventilated climate
10	Sept-Dec	Elm, Willow, Osier	Loam and slopes exposed to the North or South	
11	Oct-Dec	Hazelnut, Poplar, Elm	Rocky soils	End of June it has to rain
12	Oct-Jan	Birch, Willow		The summer has to be rainy
13	Mid Sept-mid Dec	Pubescent Oak, Poplar, Lime Tree	Muddy and sandy soils, exposed to shadow	Windy climate
14	Sept-Jan	Oak, Hazelnut		
15	Aug-Dec	Elm, Lime Tree, Oak	Wet and sandy soils	Heavy rains in July are crucial for a good season
16	Oct-Dec	Lime Tree, Elm, Birch, Poplar	Muddy and sandy soils exposed to the North	Wet climate
17	Sept-Jan	Osier, Willow, Poplar	Wet and sandy soils, rich in calcium	Rainy summer
18	Oct-Dec	Lime Tree, Elm, Hazelnut	Wet soils, far from vineyards and their chemicals/pesticides	Rainy summer

What emerges from the data is a complex, sophisticated perception of the ideal environment in which white truffles may grow. Not only are there specific preferences for particular trees, but particular soil and climatic conditions are also considered and thought to be able to enhance the occurrence of truffles and the development of their hypogeus fruiting bodies. Equally remarkable, most *trifulau* believe in the crucial role played by the moon in the growth of white truffles, whose maximum “ripening” always takes place 2-3 days before the moon “changes” (full moon).

A few study participants even reported some experiments that they conducted by intentionally planting what are considered the most suitable trees (Table 1), in order to “promote” truffle growth, in an analogous manner to what has been done for decades for black truffles (*Tuber melanosporum* Vittad.), but these attempts were unsuccessful and later abandoned.

### Traditional truffle search, storage, and the role of the dog

“*Without the dog, the man is nothing; and without the man, the dog is nothing*” (R.A., *trifulau*).

The role of the dog, and dog training, in the search for truffles is considered crucial for the final outcome of the search. Although pigs were mainly used to search for truffles during the last few centuries and partially until the 20^th^ Century in Central Italy, and even today in France, the dog has represented the core of the truffle search in Piedmont for at least several decades. All *trifulau* share the view that a close relationship between dog and collector is the foundation of the search for truffles. This connection is described as fundamental for the performance in the field and for the end result of the search.

**Fig. 3:**
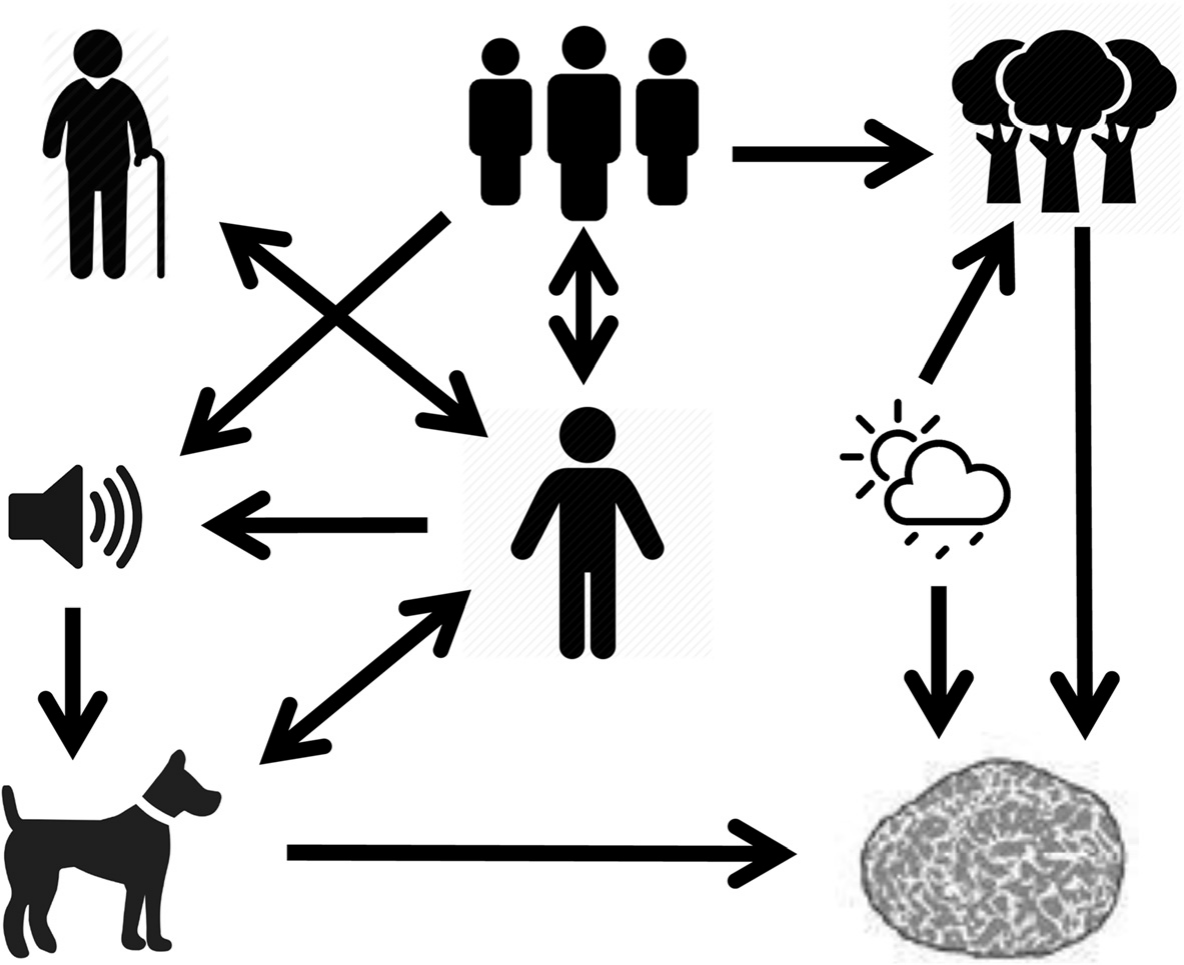
Interrelations among gatherers, TEK holders, communities, language, environments, climate, truffles and dogs

The dog in the truffle ecosystem can be described as a linking element between human beings and nature; the animal works in many cases like a “translator” for nature, providing essential information for the *trifulau*, thanks to its keen senses and extensive training. Most of the respondents grew up with “their” truffle dog, which means that their understanding of and connection to nature was taught and intensified on a daily basis for decades. The dog represents a third dimension in the relationship between men and truffles, which thus becomes triangular. In other words, the depth of the ethnozoological/ethnoethological knowledge (man-dog) seems to be crucial for the success of ethnomycological practices (man-truffle). This is unique in ethnobiology, since the link between human knowledge and truffles is mediated via an animal (dog). TEK regarding truffles therefore evolved over many decades in a complex “cobweb” involving gatherers, TEK holders, local communities, language, environments, climate, truffles and dogs (Fig. 3).

Among truffle gatherers, there has always been a lot of rivalry around their dogs and their capabilities and it was suggested that in the past some *trifulau* poisoned or killed “rival” dogs, although informants tended to downplay these incidents which they mainly consider urban legends.

While a few informants believe that specific dog breeds are more suited than others for the search for truffles (i.e. Lagotto, Breton, Collie), others believe that the breed is not as crucial as its training, which generally consists of enhancing the dog’s perception of the smell of truffles by using bread mixed with truffle oil or even directly with small (rotten) truffles every day for at least six months.

It is interesting to point out, however, that most elderly gatherers, despite the dog training courses available today, continue to want to personally train their dogs, normally using the Piedmontese dialect/language. This last point leads some informants to believe that this dimension of the intimate relationship between the *trifulau* and his dog is destined to be progressively lost, because the younger generations prefer to buy dogs already trained by specialized centres. Moreover, the language issue is a crucial one and makes the circle of *trifulau* quite isolated and completely impermeable to prospective gatherers coming from other Italian regions, or even locals, who were not born in the area. All the interviewed *trifulau* were in fact fluent in both standard Italian and in the local Piedmontese idiom.

This reminded the author of an episode he had a few years ago upon moving to Southern Piedmont, when he met a Calabrian/South-Italian migrant living close by. At some point in late autumn, this man pretended to convince the author to buy some white truffles from a Calabrian friend (truffle gatherer) living in the area, who experienced serious difficulties in selling his gathered white truffles on the local market. Although this aspect needs to be investigated further, the interviewees confirmed that the tradition of gathering truffles and the attached informal economic circuits are part of the local identity (meaning Piedmontese), which may also indicate that the likelihood of strangers (foreign migrants or even South Italians living in Piedmont) entering into the abovementioned economic circuits is probably extremely remote. As proof of this idea, traditional dog training and schooling, according to most of our informants, should be done following the rules of the local language/dialect.

Some gatherers prefer dogs with white coats, because this makes them easier to recognize and locate in the middle of the night (around 3 AM), when the search is generally conducted by most *trifulau* as noises are minimal at this time and thus the dog is believed to have fewer distractions and as a result more easily maintains its attention to the smell of truffles. The best performance is often displayed by dogs that are at least 4 years old.

In the field, there are two methods for searching for truffles: one that is suited to the lowlands, where the dog has to point to the soil from the top, and another more appropriate for hilly landscapes, where the dog has to move from the bottom to the top of the hill; as it is believed that the truffle smell “runs” 20-30 cm from the soil. After the dog has perceived the smell and then the location of a truffle in the soil, it begins to dig out the truffle, at which point the *trifulau*, with the help of a hoe, excavates the fruiting body. Afterwards, and very importantly, the hole in the ground must be completely covered using dry leaves. All of the respondents affirmed their desire to not harm the natural spore environment when digging out the fruiting bodies of truffles, as this is seen as crucial for allowing the next reproduction cycle. Moreover, some *trifulau* that were interviewed actively take part in improving external factors for truffle growth by “cleaning” the surface of potential truffle spots of rotten wood or other species that are known to be harmful to truffle growth, since they are recognized to change the humidity balance of the soil. By trying to optimize external conditions for truffle growth, *trifulau* are using practices that are also part of their TEK. 

In the past truffles were stored in clay pots filled with sand; today it is more common to use a small polystyrene box, where the truffles are wrapped with a piece of wet cloth or paper, and kept refrigerated – in this way truffles can last up to 15 days. Also, some *trifulau* used to store truffles in jars containing raw rice grains; however this practice is decreasing today, as rice tends to de-hydrate the truffles and they spoil more quickly.

Moreover, the sense of community and solidarity among *trifulau* seems to suffer; the traditions of non-written codes of conduct often no longer exist and, to quote the words of an old interviewee, “the *trifulau* today use their elbows a lot more than in the past. ” One of the *trifulau*, informant 19, described the gathering in former times as an activity of rural people who went out for truffles when the agricultural harvest season was over; in those days, truffles contributed to the subsistence income of families. Almost all interviewees emphasized that today there are many easier ways to earn money and therefore the practice is described more as a passion-driven one, where the love of nature and the joy of “finding” white truffles are fundamental.

According to the respondents, to exploit the full potential of a truffle dog, years of extensive training with an experienced *trifulau* are required, although this also depends on the capabilities of the animal. It is not unusual that well-trained dogs are sold to other *trifulau*. The informants reported that prices can go as high as 15,000-20,000 Euros for a dog with a remarkable record of finding good truffles.

Alternatively, prospective *trifulau* have the possibility of sending their dog to the truffle dog training centre (*Università dei Cani da Tartufo,* "University of the Truffle Dogs"), located in the village of Roddi, which is a much cheaper option. The dog training at this private institution lasts only three weeks. Correspondingly, this sort of *externally acquired knowledge*, which sharply contrasts to the *locallytransmitted TEK*, is widely rejected by many *trifulau* in the community and truffle gatherers with these dogs are clearly ranked on a lower hierarchical level – and they are also associated with negligent truffle hunting practices, especially if they cannot rely on a family background in truffle gathering and are not rooted in the Piedmont region.

## Discussion

### Environmental and social changes

Some *trifulau* pointed out that environmental changes have occurred in recent decades, as a result of increasingly degraded soils precipitated by wine entrepreneurs, who have not only tremendously expanded the areas devoted to viticulture but also made extensive use of chemicals, especially in the past few years.

A major concern for the *trifulau* of the Langhe and Roero areas is the reduction in the availability of *Tuber magnatum* Pico. The respondents believe that, apart from exceptional years, the amount of harvested truffles has been in decline for many decades, and estimate that over the last 40-50 years the collection has almost halved. It is believed that the main reason for this decline is the disappearance of the secondary forest owing to the expansion of viticulture and hazelnut trees planted for industrial purposes. Other factors that have been mentioned include the use of herbicides in farming systems and the abandonment of a meticulous management of the forests, where locals used to collect firewood, thereby keeping marginal land areas “clean”, which instead today are completely abandoned.

At the same time, however, many *trifulau* believe that climate change may be in part responsible, as summers are much warmer and drier than in the past. In the perspective of a few gatherers, climate change seems to claim a lion’s share of the blame in the slow, yet continuous, disappearance of the white truffle. As most respondents pointed out, warmer and drier springs and summers lead to a drop in the subterranean water level, which dries out the soil, thus taking away one of the most important growing conditions for white truffles: a balanced humidity level. Moreover, floods are also indicated as major threats to the maintenance of ideal soil conditions for the growth of white truffles. All *trifulau* agreed that the large flood which occurred in the area in 1994 devastated the occurrence of white truffles in the following years. One consequence of these trends is that today, much more than in the past, the optimal areas for searching for white truffles are limited, and consequently the search is more “intense” and is regularly done nowadays using cars and moving quickly from one potential suitable area to another. In today’s world where electronic devices are ubiquitous, sometimes the search for truffles is facilitated by electronic geo-localisation gadgets. The search is mainly done only in selected locations considered worthwhile, the duration of the search is compressed, and long walks are avoided, and as a result the deep *embeddedness* between gatherers and their *oikos* is mostly lost.

The impact of such changes are multidimensional; first of all, owing to the fact that there are far fewer truffles available, the monetary impact of truffle gathering nowadays can be described as an auxiliary income, whereas it used to be a more lucrative “profession” according to the respondents. Some gatherers confirmed, however, that even though the monetary importance of truffle gathering has decreased significantly, their motivation to search for truffles is driven more by a genuine passion for nature and the interaction with their dog than by the monetary perspective alone. Also, as already pointed out above, the connection to their “home-territory” is becoming weaker as *trifulau* have to increase their hunting area by using a car as a means of transport.

Another difficulty mentioned in connection with the decline of truffles involves the limited opportunities for the training and exercise of truffle dogs. With fewer truffle spots, training is becoming noticeably more difficult. Fewer truffles also mean fewer moments of success for the prospective truffle dog, which leads to faster demotivation. Therefore, the training of truffle dogs takes more time and effort for the *trifulau*. One informant stressed the fact that rising competitive pressure between gatherers, combined with fewer truffles, has the negative effect that the white truffle is harvested too early. Although only very well-trained dogs are actually able to smell immature truffles, this seems to make the situation worse as “green”, unripe truffles (as they are called by some informants) do not have time to release enough spores into the soil to prepare the ground for future truffle growth at the same location.

Moreover, the growth of this human pressure on a decreasing number of truffles could lead to a much more laborious search, with the unavoidable frustration of the dogs, which would in turn call for much more intensive dog training techniques. All in all, and in contrast to that recently observed in Italy among orchardists [34], the ability of truffle TEK holders in Piedmont to adapt to climatic changes seems very limited.

Apart from purely environmental changes, the community feeling and solidarity between the gatherers also seem to suffer damage in the process described above. Although many gatherers stressed the fact that they used to have, in general, little motivation to get in contact with other *trifulau*, the customary *code of conduct* described above shows that members of this community used to treat each other with respect and goodwill.

The aforementioned findings further confirm the urgent need for a multi-disciplinary analysis that combines diverse data in order to properly investigate climate change and environmental changes, as other works have recently highlighted [35].

### Transmission of TEK and its changes

The majority of the informants learned truffle gathering during their childhood from their fathers or grandfathers, and their families have been involved in gathering for at least three generations. However, there are exceptions: informant 20 for example has not got a family background and learned truffle gathering in his adulthood from his friend informant 21

These two different trajectories lead to two methods of training that are further described as the vertical transmission of TEK for the former and the horizontal transmission of TEK for the latter.

In the first one, the child is guided in the woods by the older generation over a certain period of time. For example informant 22 started learning at 6 years of age and went for the first time on his own when he was only 12 years old. The older generation passes knowledge to the younger generation by practicing together following the learning concept of “observation – understanding – doing”. The first thing to learn are the truffle locations and then how to work with the dog. Furthermore, there is a kind of code of conduct on how to behave during the search as well as to respect nature in order to maintain the culture. This *code of conduct* basically instructs how not leave litter in the woods, pick grapes or step on a fresh field.

The horizontal transmission of knowledge, apart from occurring peer-to-peer between adults, mainly differs from the previous one in the following points: the experienced gatherer does not tell the adult learner the locations of truffles and there are just a few joint hunts to explain the basic techniques. Also, the adult learner goes by himself with an experienced dog.

In general the latter practice is considered very rare as the sample group confirms, and the predominant method is still the transmission of TEK within families.

The gatherers informant 19 and informant 23, who do not have descendants to whom they could pass their knowledge, are very sceptical about teaching someone from outside the bounds of the family. They will only teach someone who is deeply interested and has the strength to stick with it. If they cannot find anyone like this they would rather let their knowledge be lost. Informant 19 describes the change in the new generation in which young people go to the city and lose the connection to their region as one reason why the gathering culture in the family is in danger.

Nevertheless, both ways are based on TEK that is passed from one person to another or generated during their shared experiences in the woods. At the same time, however, many informants today admit to enriching their knowledge through books on truffles and other sources, especially information online. What was described at the beginning of this paper as TEK is, in reality, configured as a complex conglomerate of traditional knowledge mixed with other standardized knowledge from the media. Some of the respondents also complain about what they perceive as a decline of interest in truffle gathering by the younger generations, who are often portrayed as only interested in the mere cost aspect of collection.

In particular, the transmission of the subtle TEK concerning the places, the *oikos*, where truffles grow seems to fade and the transmitted and acquired knowledge is reconfigured as a simple mosaic of techniques, in which a deep understanding of the environmental dynamics is diluted. This phenomenon of collection “optimization” is not only the operating practice of truffle collection, but also, as our unpublished data confirms, the trend in the current trajectories of urban *foraging* of wild food herbs in Western Europe, often promoted via training courses in the field and popularized by diverse chefs of contemporary cuisine.

Moreover, a few respondents pointed out the common practice of assigning an experienced dog to an unexperienced truffle gatherer to accelerate the learning process between the two; in this way, the well-trained dog supports the learning experience of the untrained gatherer. Through precise observation of the dog’s behaviour and the effects of his own actions, the prospective *trifulau* increases his awareness and sensibility towards the animal. Thus, not only can the experienced truffle gatherer be regarded as a knowledge holder but also the trained dog that is playing a crucial part in the transmission of TEK to the next generation of *trifulau*.

The transmission of knowledge in the culture of truffle gatherers in Piedmont therefore appears to be shaped in four dimensions: from human to human (older generation teaching the younger generation, or peer-to-peer), from human-dog (training of dogs by experienced *trifulau* or dog trainers), from dog to human (transfer of knowledge from experienced dog to prospective gatherer) and, presumably to a small extent, from dog to dog (two gatherers with their dogs). The latter was reported to occur from time to time, especially when two generations of one family with their own dogs are hunting together. The act of two friendly *trifulau* joining for a truffle search can be considered a rare event with regard to the increasing competition in the forest.

Finally, one of the popular recurring symbols in the local mythology of Langhe and Roero is the gatherer’s “book”, in which the truffle gatherer documents with pictures his knowledge, especially potential truffle locations in his territory. According to this myth, the experienced *trifulau* takes the book with him to his grave instead of passing it on, even to his closest relatives. The gatherers met in this study, however, did not use a book to document truffle locations or their performances. One of the *trifulau* interviewed takes notes about the date, place and size of the truffles found, which enables him to make a rough prediction concerning the next time it may be worthwhile to visit that spot again. Some of the interviewees did not adhere to the stereotype of the secretive *trifulau*, and tended to show that information regarding TEK on truffles has to be generously shared.

Yet, it does not seem to be self-evident that potential inheritors of this knowledge are always present. Only two of the six informants interviewed in-depth have already transferred their knowledge to someone else: one gatherer passed it on to his son (who is also part of the study sample and young enough to pass it on to potential descendants), and the other one to a friend who is also a gatherer; whereas two of the *trifulau* see almost no chance of handing down their TEK to someone else in their family as they have no children and other relatives (e.g. nephews) are not interested in the activity. Even the transmission to prospective *trifulau* outside of the family was seen as unlikely by the informants, as most young people, they argue, would prefer to live in the city and the exhaustive work of hunting for truffles in the middle of the night is not perceived as something desirable within this generation. Finally, some of those gatherers who have already transferred their knowledge to others are not confident that the transmission of TEK inside the *trifulau* family will play the important role that it used to.

## Conclusion

The recorded ethnomycological knowledge is deeply embedded in the bio-cultural heritage [36] of the Langhe and Roero areas. This heritage, however, reveals a contradictory picture: on one hand, this complex web of knowledge, beliefs and practices, is the result of at least a 300 year-long co-evolution between man and nature within a given territory that still survives, albeit eroded; on the other hand, different environmental, economic, and social dynamics seem to threaten this heritage.

The author believes that the role that the local gastronomy has played for a few decades in the area could become a key turning point for sustaining both the environment and the TEK related to white truffle gathering. However, the promotion of local foods does not necessarily lead to its survival. It may even be assumed that the luxury status that the Piedmontese white truffle has in the worldwide food specialities market may isolate it from the territory and the people living there so that there may not be a deep motivation to preserve it. Most of our informants, in fact, beyond their awareness of the decreasing availability of truffles due to ecological change, were not particular conscious of which possible solutions could be enacted'. The truffle business is at risk because of expanding vineyards, but at the same time the increasing wine and food tourism industry as well as the stress of local stakeholders on *sustainable* gastronomies [37] – this in the area in which the international *Slow Food* movement was born in 1989 – could promote awareness for a heathy environment, in which white truffles can survive, grow, and also be sustainably gathered.

Further investigations should be conducted with the aim of analysing the opinions that the local communities (apart from *trifulau*) in the Langhe and Roero areas have regarding not only the conservation of the natural habitat of the white truffle, but also who should care for them and who could take initiative for this.

As already observed in a completely different context – the post-Communist harvesting of diverse wild medicinal plants on the Albanian side of the Korab Mt. for trade [38] – a hyper-commodification of natural resources could lead to the loss of a reasonable perception of the limitation of the same resources. Therefore, since keeping a dynamic but balanced equilibrium between gatherers and wild resources is crucial for avoiding overexploitation, this could be promoted by fostering a more continuous and long-term interaction (via environmental educational frameworks) between local communities and nature. This is probably what ultimately would be able to reinforce socio-ecological resilience in the local management system [39]. Local appreciation for the sustainable use of natural resources is thus a crucial issue for their management and conservation, especially when faced with external market pressure; on the other hand, conservation knowledge can develop through a combination of ecological understanding and learning from crises and mistakes [40].

A possible intriguing and challenging opportunity may be anticipated in the way white truffle consumption is communicated within the gastronomic world. Instead of focusing only on the sensory/aesthetic (mainly smell-based), high-quality value of this food item, it may be important to re-connect consumers to the environment, and make clear that the occurrence of truffles in the South-Piedmontese cuisine is related to a specific bio-cultural landscape [41], which in turn is the result of a long co-evolution and negotiations between locals and their “nature”. In South Piedmont some truffle gathering courses and workshop are sometimes offered to tourists, but this generally happens within a framework where the core is *spectacularization* of the search and discovery of the “magic” truffle.

In other words, a sustainable gastronomy focusing on the *sense of place* as performative experience [42], instead of on eccentric, luxury, smell- or taste-based events, could potentially contribute to a shift in the experience among consumers and *foodies*. Finally, the South-Piedmontese civil society should probably also reconsider the exaggerated contribution that the high-quality, market-driven wine industry makes in the local economy and begin to develop routes of sustainability within the local foodscapes, where *trifulau*, dogs, and the environment could continue to foster the inextricable ethnoecological cobweb woven over centuries of mutual interactions.
